# A cross-sectional study of Canadian children's valuation of literacies across social contexts

**DOI:** 10.3389/fspor.2023.1125072

**Published:** 2023-03-16

**Authors:** Emily Bremer, Philip Jefferies, John Cairney, Dean Kriellaars

**Affiliations:** ^1^ School of Kinesiology, Acadia University, Wolfville, Nova Scotia, Canada; ^2^ Resilience Research Centre, Dalhousie University, Halifax, Nova Scotia, Canada; ^3^ School of Human Movement and Nutrition Sciences, University of Queensland, Brisbane, Queensland, Australia; ^4^ College of Rehabilitation Sciences, University of Manitoba, Winnipeg, Manitoba, Canada

**Keywords:** physical literacy, subjective task value, movement, physical activity, education

## Abstract

**Background:**

Children, on average, do not engage in sufficient physical activity to reap the physical, mental, and social health benefits. Understanding the value that children place on movement across social contexts, and the relative ranking of this valuation, may help us to understand and intervene on activity levels.

**Method:**

This exploratory study examined the valuation of reading/writing, math, and movement across three social contexts (school, home, with friends) among children 6–13 years of age (*N *= 7,845; 51.3% male). Subjective task values across contexts were assessed with the valuing literacies subscale of the PLAYself. One-way Kruskal-Wallis ANOVAs were performed to test for differences between contexts and between literacies, respectively.

**Results:**

Sex differences and age-related variation were explored. Valuations of reading/writing (*d *= 1.16) and math (*d *= 1.33) decreased across context (school > family > friend), while the valuation of movement was relatively stable (*d *= 0.26). Valuations differed substantially with friends (*p *< 0.001, *d *= 1.03). Sex dependent effect sizes were minimal (*d *= 0.05–0.11).

**Conclusions:**

Movement is highly valued by children across social contexts; thus, programming across contexts should be prioritized to align with their valuation.

## 1. Introduction

Regular participation in movement experiences during childhood are associated with a range of positive physical, mental, and social health outcomes (1–4), yet the physical activity levels of children remain low (5, 6). Thus, it is essential that we continue to explore factors associated with participation in physical activity to effectively intervene in this area. Physical literacy is an increasingly popular framework by which to understand, guide and foster participation in and across diverse movement contexts (7). Physical literacy can be viewed as the convergence of the motor, affective, social, and cognitive factors that are related to sustained participation in physically active pursuits (8). Cairney and colleagues' conceptual model of physical literacy demonstrates how physical literacy is indirectly associated with physical, mental, and social health outcomes through participation in physical activity, while acknowledging the moderating effect of both individual factors (e.g., sex, age) and social/environmental context (e.g., home, school) on these pathways (8). In addition to movement competence, core psychosocial components of physical literacy are an individual's motivation and confidence to engage in an activity (9). Motivation and confidence in turn are also closely related to the value one attributes to an activity, which is known as subjective task value (10). Eccles et al. propose that subjective task value incorporates one's interest in or enjoyment of the activity; their perceived importance of being good at the activity; their perceived usefulness of the activity; and their perceived cost of engaging in the activity (10). Ultimately, subjective task value influences an individual's decision to engage in a given activity, the amount of effort they expend on the activity, and their actual performance on the activity (11).

Understanding the value that children place on movement may help to further appreciate their level of engagement in physical activity through this connection to motivation. Previous research on subjective task value among children and adolescents has largely focused on academic subjects and sports (10, 12, 13). This body of work suggests that sports are generally valued more highly than academic subjects, such as math and reading (12). Moreover, males tend to place a higher value on sports than females, while females place a higher value on math and reading than males (12, 13); these differences are likely due to societal gender norms, not actual biological sex differences. A limitation of much of the extant literature on valuations is the emphasis on limited forms of participation, sport in relation to movement, and narrow contexts, specifically, the school environment. Sport for example is only one very specific form of participation; children engage in a diverse range of movements, some of which are highly structured and organized, others are open and discretionary or self-guided. Movement contexts can be competitive (as in the case of sport) or not (as in the case of activities like dance or circus). Children are also immersed in a diverse range of social contexts (e.g., with peers at school, at home with family, and with friends, among others). Understanding the valuation of activities across different contexts has only recently been explored. For example, Houser and colleagues (14) recently noted that children's valuation of movement was significantly greater than reading, writing, and math across the contexts of school, home, and with friends during the COVID-19 pandemic; and that decreased physical activity during the pandemic occurred in tandem with a decreased valuation of movement across these three social contexts.

The comparators across contexts are especially interesting given that so much of movement occurs outside of school, and children and youth spend most of their time in school doing activities related to other forms of literacy. Moreover, while sex differences in the subjective valuation of sport compared to math and reading have been found between males and females, we know of no studies that have examined relative differences in subjective valuations of literacies by sex, across different contexts. The purpose of this exploratory study was to examine the valuation of reading and writing, math, and movement across three social contexts (school, home, and with friends), while testing for sex differences and age gradients in these valuations among Canadian elementary school-aged children.

## 2. Method

### 2.1. Design and sample

This cross-sectional study utilized an extraction of the first year of data (collected in 2016) from a longitudinal study of the physical literacy of Canadian children and youth. The STROBE checklist for cross-sectional studies was employed (15). The sample included 7,845 individuals aged 6–13 years (M = 9.20, SD = 1.85) from three Canadian provinces. There were approximately equal proportion of males (51.3%) and females (48.7%). Given the constraints of working within the school system, no additional demographic data was collected. All participants completed the PLAYself tool during the school day. The study was approved by the University of Manitoba Health Research Ethics Board (Approval #: H2012:077 and H2016:254) Prior to participation, child assent and parental informed consent were obtained.

### 2.2. Measures

PLAYself is a brief, self-report measure of physical literacy for children and youth. The PLAYself tool has three unidimensionally distinct, ordinally ranked sub-scales related to environmental competency and participation, physical literacy self-description and valuing literacies (16, 17). The valuing literacies section was used to assess the subjective task values of reading and writing, math, and movement across the contexts of school, home, and with friends. For this study, only the nine literacy task values questions were included in the analyses. For the valuing literacy subscale, participants were asked to indicate their agreement of the importance of each of the three literacies, in three different contexts. The three question stems were “*Reading and writing are very important*”, “*Math and numbers are very important*”, and “*Movement, activities and sports are very important*”. These three stems were each followed by three contexts including, “*in school*”, “*at home with family*”, and “*with friends*”. The nine items were answered on a 4-point ordinal scale ranging from 0 (strongly disagree) to 3 (strongly agree); however, previous validation work indicated that these items should be rescored from 0123 to 0012 (17). Therefore, the current study employed the 0–2 scoring, where “strongly disagree” and “disagree” were collapsed into one response, as recommended by Jefferies and colleagues. The single item scores for each literacy (i.e., reading/writing, math, and movement) across each of the three contexts (i.e., school, home, and friends) is used to indicate the valuation of that literacy by context. A similar approach has previously been used by Houser and colleagues (14). The PLAYself tool as a whole has demonstrated good psychometric properties including satisfying the requirements of Rasch analyses, exhibiting internal reliability, excellent test-retest reliability, and good convergent validity with the domains of physical literacy (17). The valuing literacies subscale, specifically, has demonstrated unidimensionality and good internal consistency (17). Moreover, the overall internal consistency of the valuing literacies subscale for the current study was good (Cronbach's alpha = 0.86), and the internal consistency across literacies and contexts, respectively, was acceptable (Cronbach's alpha range = 0.70–0.80).

### 2.3. Analyses

To explore valuations of the different literacies across the three contexts, one-way Kruskal-Wallis ANOVAs were performed. In the first three analyses, for each literacy, we sought to check whether valuations differed between contexts (e.g., reading/writing in school compared to at home and with friends). In the second three analyses, this was then switched to examine whether valuations in each context differed between literacies (e.g., scores in school for reading/writing compared to math and movement). Post-hoc comparisons were made using Wilcoxon rank sum tests (with continuity correction). To determine potential differences between males and females in valuations, we analysed scores using Wilcoxon rank sum tests with continuity correction with Tukey's HSD. Ordinal regression was employed to examine age-related variation in valuations. Effect sizes are reported as Cohen's *d* with 0.2, 0.5, and 0.8 indicating small, medium, and large effects, respectively (18). All analyses were performed in R v4.0.0 (19) using R Studio (20) and jamovi (The Jamovi Project, v1.6.14). An alpha level of 0.05 was deployed.

## 3. Results

Table 1 reports the differences in valuations between literacies and between contexts. Large effect sizes (*d* = 1.16 and 1.33) were revealed for the observed reductions in valuations of reading/writing and math from school to family to friend contexts. In contrast, there was a small effect (*d* = 0.26) detected for valuations for movement between contexts. Within context comparisons between literacies revealed significant and substantial differences (*p *< 0.001, *d* = 1.03) in valuations in the friend context.

**Table 1:** Overall and pairwise differences between literacies and contexts (Mean, SD). Effect sizes are listed for significant overall differences.

Literacy		Context		H (df)	Effect Size	Pairwise comparison differences
					*d*	
	*School*	*Home*	*Friends*			
*1. Reading/writing*	1.51 (.58)	1.25 (.66)	1.01 (.74)	1969.2 (2)*	1.16	Between all*
*2. Math*	1.56 (.57)	1.24 (.68)	.98 (.76)	2428.6 (2)*	1.33	Between all*
*3. Movement*	1.48 (.60)	1.36 (.65)	1.41 (.64)	129.08 (2)*	0.26	Between all*
H (df)	70.47 (2)*	157.35 (2)*	1647.1 (2)*			
Effect Size (*d*)	0.19	0.28	1.03			
Pairwise comparison differences	Between all*	Between 1 and 3*, 1–2, and 2–3*	Between 1 and 3*, 1–2, and 2–3*			

* *p *< .001.

**Figure 1:**
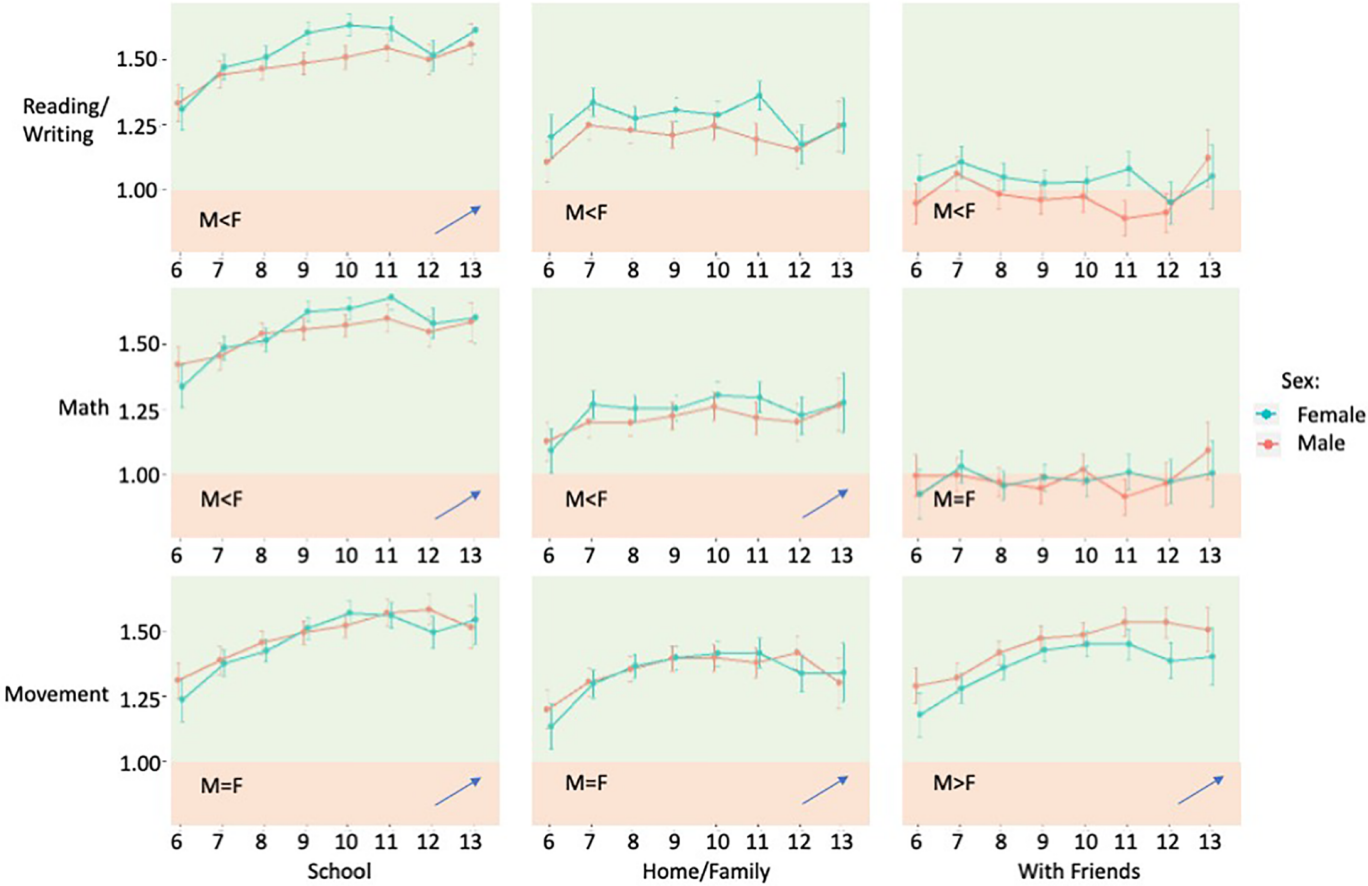
Literacy valuations (Mean, SD) in each context separated by age (bottom axis, years) and sex (M = red, F = green). Green shading corresponding to agreeable valuations, red to disagreeable. Overall sex differences for each cell indicated (W, *p *< 0.05). Age gradients indicated by slope (blue arrow, ordinal regression with age, Kendalls tau-b).

Table 2 reports the sex dependent literacy valuations across the three contexts. Overall, the sex dependent effect sizes were minimal (*d* = 0.05–0.11) with a noted reversal of effect for the movement context with friends (M > F) relative to those evident for reading/writing and math (F > M). Figure 1 reports the age gradients and sex differences in valuation of literacies across the three contexts. A significant age effect is evident across all three literacies within the school setting, with valuations increasing with age. At home, valuations of math and movement, but not reading/writing increase with age. Only valuations of movement increase with age while with friends.

**Table 2:** The literacy valuations (Mean, SD) at school, home and with friends separated by sex. Effect size is reported for significant differences.

Literacy	Context	Male	Female	W	Effect Size
				(*p* value)	*d*
Reading/Writing	School	1.48 (.59)	1.55 (.56)	7251896 (<.001)*	0.11
	Home	1.21 (.68)	1.29 (.64)	7251220 (<.001)*	0.11
	Friends	.97 (.75)	1.04 (.73)	7295150 (<.001)*	0.10
Math	School	1.54 (.58)	1.58 (.56)	7475726 (.014)**	0.06
	Home	1.22 (.71)	1.26 (.66)	7482594 (.025)**	0.05
	Friends	.98 (.77)	.99 (.74)	7645312 (.648)	
Movement	School	1.48 (.61)	1.48 (.59)	7764970 (.385)	
	Home	1.36 (.66)	1.36 (.65)	7684612 (.969)	
	Friends	1.45 (.64)	1.38 (.64)	8107299 (<.001)*	0.11

Sex differences.

* Significant at *p *< .001.

** Significant at *p *< 0.05.

## 4. Discussion

This study examined children's valuation of reading/writing, math, and movement across three social contexts and the results demonstrate differences in contextual valuing of these literacies. Large differences are present between the valuing of movement and reading/writing, and math while with friends, where we see movement valued and reading/writing and math devalued or neutrally valued. The equal valuation of movement across social contexts, and its relative valuation to reading/writing and math, suggests that movement may represent a powerful opportunity to aid in children's agency for health and well-being, as well as educational success. A child's agency can be defined as their “capacity to act deliberately, speak for oneself, and actively reflect on their social worlds, shaping their lives and the lives of others” (21). Providing opportunities for children to engage in activities that they value, such as movement, may contribute to their sense of agency and also aligns with Article 12 of the United Nations Convention on the Rights of the Child (22). The power of movement has been further highlighted within the concept of physical literacy to inform public health, recreation, and educational policies where providing multiple movement contexts is considered a key pillar of effective policy in these areas (7).

We found that while children value movement highly in school, this valuation is slightly lower than the value they place on reading/writing and math in school. Further, we see a devaluation of reading/writing and math across contexts from school to home to friends. It is possible that children place a higher valuation on reading/writing and math at school and at home because they know that their teachers and parents value these tasks, potentially moderating children's valuations of these literacies. The fact that valuations at school increase with age also suggests an increasing awareness of what “should” be valued as children get older. In contrast, only valuations of math and movement increase with age in the home setting; and only valuations of movement increase with age when with friends. This finding is in contrast to previous literature that has found subjective task values of math, language arts, and sports generally decline as children get older (12, 13). It is unclear why we are seeing these age-related differences in valuations in our data, but they are possibly due to increasing teacher and parental influence on children's valuations.

Likewise, movement at school is predominately teacher-directed through physical education classes and movement breaks. This slight devaluation of movement at school, relative to the other literacies, is interesting. Movement is essential for the physical and cognitive health of children (1–3); however, traditional academic subjects are often labelled as “cognitive” or more important, while movement has been relegated to a “non-cognitive” or less important subject. This positioning is erroneous and devalues movement as important for cognition. While the benefits of movement on cognition have been established, one must also consider that movement itself requires cognition (23). Previous research has demonstrated the utility of movement for cross-curricular linkages in school, through for example movement infused academic lessons (24, 25) and physically active classrooms (26). We must not only consider the benefits of “adding” movement to more traditional academic work, but how literacies such as reading/writing, and math are already naturally incorporated into many movement-based activities in which children participate (e.g., keeping score during a game, and explaining novel game ideas and rules to friends and playmates). Future work may explore how delivering physical literacy-enriched curricula at school can provide opportunities to incorporate movement throughout the day while encouraging greater valuing by educators and families. Moreover, there is a need to work with adults (e.g., parents, teachers) and children in fostering a role of importance of all literacies across social settings.

Similar to previous research, our results indicate that small sex differences are present in children's task values (13); however, novel to this study is the pattern of these differences by context. Females valued reading/writing and math more highly than males in school and at home; and valued reading/writing more highly when with friends. We postulate that these differences are due to societal gender biases, rather than actual sex differences, which warrants future study into this area including the influence of friends (of similar and different sexes and genders) on differences in valuation. There were no sex differences in the valuation of movement in school or at home. However, when among friends, males valued movement more highly than females. It is worth further exploring why sex differences in movement only appear with friends and not in the other contexts. Previous research on children's agency in social contexts has found that children perceive themselves to have the most agency when with their friends, in comparison to their parents or teachers (27). It is possible that societal gender biases are creating sex-based differences in this context. More research is needed to further explore the role of sex and gender in children's valuation of movement with friends. It is relevant to note that males' higher valuing of movement with friends aligns with known sex differences in participation in physical activity (28). The fact that sex differences in the valuing of movement only exist among friends, and not at school or at home, highlights an important opportunity for strategic intervention and an opportunity to combat sex and gender bias as it relates to movement. For example, physical education teachers can help to emphasize the importance of movement for all individuals while incorporating novel games and activities into their classroom to ensure no inherent sex or gender biases are present in the activities. Further, community organizations can support females to be more active outside of school with their friends through fun and engaging female-oriented physical activity and social programming.

The findings of this study are strengthened by its large sample and assessment of the value placed on literacies across three social contexts; however, they are not without their limitations. PLAYself was not designed as a measure of subjective task value, rather as a measure of perceived physical literacy. However, the questions regarding the valuing of literacies provide us with an opportunity to explore one aspect of subjective task value across multiple social contexts. While common measures of subjective task value ask about multiple facets of the construct, PLAYself asks children to respond to questions that ask “[Reading and writing/Numeracy/Movement] is very important at [school/home/with friends]”. These single-item questions could be considered a measure of either attainment or utility value, depending on how the child interprets the question; however, it does not disentangle the construct of subjective task value, nor does it disentangle valuations in different movement contexts (e.g., sport, dance, free play, etc.). Further, while these single items have previously been used as a measure of task valuations (14), it is unknown how well they represent the construct of subjective task value. Lastly, the data included in this study were collected in 2016. It would be important to replicate the analyses with current data to see how (if at all) these valuations may have shifted since then. Despite these limitations, our results are consistent with other multi-item scales of subjective task value as they relate to sex differences (12, 13).

The results of this study highlight the value that children place on movement across social contexts. These findings point to possible interventional approaches that can be explored in future work including the role of movement for childhood agency, physical activity, and educational success. Future work should also seek to understand why sex differences are present in valuations across contexts and work to mitigate these differences. Understanding how subjective task values of movement vary by context, through the lens of physical literacy, may help researchers and practitioners to better align physical activity-based programming with children's values.

## Data Availability

The raw data supporting the conclusions of this article will be made available by the authors, without undue reservation.
